# Postural Stabilization Differences in Idiopathic Parkinson’s Disease and Progressive Supranuclear Palsy during Self-Triggered Fast Forward Weight Lifting

**DOI:** 10.3389/fneur.2017.00743

**Published:** 2018-01-22

**Authors:** Stefan Kammermeier, Lucia Dietrich, Kathrin Maierbeck, Annika Plate, Stefan Lorenzl, Arun Singh, Ahmad Ahmadi, Kai Bötzel

**Affiliations:** ^1^Ludwig-Maximilians-Universität München, Neurologische Klinik und Poliklinik, München, Germany; ^2^Abteilung für Allgemeinchirurgie, Kliniken Ostallgäu-Kaufbeuren, Kaufbeuren, Germany; ^3^Klinikum der Universität München, Klinik für Anästhesiologie, München, Germany; ^4^Abteilung für Neurologie, Krankenhaus Agatharied, Hausham, Germany; ^5^Department of Neurology, University of Iowa, Iowa, IA, United States

**Keywords:** idiopathic Parkinson’s disease, progressive supranuclear palsy, posture, posturography, falling, reafference, self-triggered disturbance

## Abstract

Progressive supranuclear palsy (PSP) and late-stage idiopathic Parkinson’s disease (IPD) are neurodegenerative movement disorders resulting in different postural instability and falling symptoms. IPD falls occur usually forward in late stage, whereas PSP falls happen in early stages, mostly backward, unprovoked, and with high morbidity. Self-triggered, weighted movements appear to provoke falls in IPD, but not in PSP. Repeated self-triggered lifting of a 0.5–1-kg weight (<2% of body weight) with the dominant hand was performed in 17 PSP, 15 IPD with falling history, and 16 controls on a posturography platform. PSP showed excessive force scaling of weight and body motion with high-frequency multiaxial body sway, whereas IPD presented a delayed-onset forward body displacement. Differences in center of mass displacement apparent at very small weights indicate that both syndromes decompensate postural control already within stability limits. PSP may be subject to specific postural system devolution. IPD are susceptible to delayed forward falling. Differential physiotherapy strategies are suggested.

## Introduction

Idiopathic Parkinson’s disease (IPD) and the most frequent atypical Parkinsonism syndrome progressive supranuclear palsy (PSP) are neurodegenerative syndromes with postural instability and different types of falling, indicating different neurophysiological etiology.

In advanced stages of IPD patients exhibit frequent forward falling, either by failure to initiate a walking motion, freezing or out of a forward motion by failure to stop. Stooped posture with forward shift of body mass can result also in falls while standing: by bending over too far or by lifting an unexpectedly heavy weight. Less frequently, falls occur in different directions. However, also small weights were reported by patients to cause falls. Orthostatic dysfunction and frontal executive disorders may contribute additionally. Early stages rarely experience falling and symptoms respond well to Levodopa, whereas late-stage postural instability is mostly Levodopa-resistant (1–5).

Progressive supranuclear palsy preferentially targets the midbrain area with vertical gaze disorders typical for the disease. All symptoms including postural instability respond poorly to dopaminergic medication and falls occur within the first year of disease manifestation (6, 7). Falls are typically unprovoked backwards without reflexive countermeasures, leading to injuries mostly on the back of the head with considerable morbidity (8) and even mortality. PSP falls are anamnestically related to “miniscule floor unevenness” or even no apparent reason at all, yet not especially during object handling (5–7, 9, 10).

Upright stance control needs to be robust against internal instability while remaining flexible to adjust against external forces (11). Visual, vestibular, and proprioceptive inputs are integrated into a multisensory concept of body in space (12). For compensation of self-initiated movement of the body in space, especially with additional weight attached to them [e.g., Ref. (13–15)], motor control relays an efference copy of imminent motion type, velocity, and expected mass displacement. Anticipatory postural adjustments (APAs) before the onset of the self-induced disturbance (16–19) precede closed-loop compensatory postural adjustments [CPAs, (19, 20)], which require at least 30-ms latency. APAs scale to the weight shift proportional to body weight and can be recorded consistently beyond weight loads 5–10% of body weight (15, 19). A central “tonic excursion limiter” provides step initiation for a wider support surface, should APA and CPA together fail to compensate perturbation as an additional safety mechanism (11).

Previous research on postural deficits in IPD indicated inadequate sensory reweighing toward higher visual and lower proprioceptive input (2, 21) and an excessive postural correction of stance to disturbing stimuli (16, 22). Postural studies in PSP are few [e.g., Ref. (9, 10)] and none of these have put object interaction in focus. The exact pathophysiological mechanisms behind PSP falling are not fully understood.

This study investigated the postural responses of advanced stage IPD with frequent falls and ambulatory PSP during self-triggered lifting [e.g., Ref. (13)] of a relatively small weight (<2% of body mass) to investigate different CPA strategies on a static posturography platform. This setup simulated a frequent daily activity, in which IPD with falling tendencies report stability difficulties including falls, whereas PSP do not.

## Materials and Methods

### Subjects

Three groups of subjects were recruited for a series of studies in IPD and PSP. Table 1 (a–c) summarizes biometrics and clinical scores. All participants gave written informed consent and data was pseudonymized at inclusion, in accordance with the Helsinki Declaration and the local ethics committee (decision 142/04).

**Table 1:** Clinical parameters of participants in this study: (a) controls subjects (CTR), (b) idiopathic Parkinson’s disease (IPD), and (c) progressive supranuclear palsy (PSP) are shown with sex (0 female, 1 male), age (median ± SD: CTR 60 ± 9.4, IPD 70 ± 7.5, PSP 68 ± 3.6), height in cm, barbell (weight of adjustable instrument, either 0.5 kg or 1 kg), disease duration (YEARS diagnosed with disease in IPD median ± SD 10 ± 4.5 and months in PSP 5 ± 6.4) and clinical scores; Unified Parkinson’s Disease Rating Score (UPDRS) with items I, II, III (median ± SD: IPD 18.5 ± 6.9, PSP 13 ± 3.2) and modified III (scaled each question/task 0–4), PIGD, Hoehn & Yahr Scale (H&Y under optimal medication, median ± SD: IPD 2.5 ± 0.7, PSP 2.5 ± 0.3); for PSP apply specifically: Berg Balance Scale (BBS) (48 ± 0.8), Golbe Score (26 ± 8.3), PSP-staging scale (2 ± 0), the scale of the NNiPPS study (Neuroprotection and Natural History in Parkinson’s Plus Syndromes, 26 ± 5.7), frontal assessment battery (FAB) (14 ± 2.9), Mini-Mental State Examination (MMSE) (29 ± 1.6), PSP rating scale (PSPRS) (30.5 ± 7.5), Schwab & England activities of daily living (SEADL) (80 ± 11), and Montgomery–Åsberg depression rating scale (MADRS) (12 ± 6.7).

(a)					

	Sex	Age	Height	Weight	Barbell
CTR1	1	60	173	70	1
CTR2	0	51	179	73	1
CTR3	1	60	163	66	1
CTR4	0	60	154	60	1
CTR5	0	67	168	64	0.5
CTR6	1	46	168	105	1
CTR7	0	40	167	90	1
CTR8	0	73	155	57	1
CTR9	0	61	168	57	1
CTR10	1	60	174	81	1
CTR11	0	56	159	65	1
CTR12	1	69	176	100	0.5
CTR13	0	60	176	81	1
CTR14	1	67	180	103	1
CTR15	1	42	183	95	1
CTR16	1	61	183	115	1

(b)															

	Sex	Age	Height	Weight	Barbell	Disease	UPDRS I	UPDRS II	UPDRS III	mod. III	PIGD	Extremities	Neck	Hoehn & Yahr	Levodopa
IPD1	0	70	170	79	1	8								1	1
IPD3	1	71	168	62	1	4	4	5	12	7	5	1	1	3	1
IPD4	1	46	175	108	1	4	0	2	16	9	0	1	1	1	1
IPD5	1	66	177	77	1	4	3	14	18	12	5	1	0	2.5	1
IPD6	1	72	170	72	1	15	2	21	19	10	6	1	0	3	1
IPD7	1	72	176	81	0.5	9	3	15	34	20	9	3	3	2.5	1
IPD8	0	73	157	65	1	12	1	16	24	14	4	1	0	2	1
IPD11	1	66	180	75	1	10	2	9	9	7	3	1	0	2	1
IPD13	0	69	168	69	1	6	2	12	7	7	3	1	0	1.5	1
IPD14	0	72	167	60	0.5	10	2	29	20	16	15	1	0	3	1
IPD15	1	63	179	66	1	18	2	20	14	13	12	0	0	3	1
IPD16	0	72	154	60	1	3	3	16	26	17	6	1	0	3	1
IPD17	1	72	167	48	1	15	2	14	19	12	6	1	0	2.5	1
IPD19	0	55	175	75	1	11	4	13	21	15	6	1	0	3	1
IPD20	0	66	162	62	1	10	0	10	18	13	6	1	0	3	1

(c)																									

	Sex	Age	Height	Weight	Barbell	Disease	UPDRS I	UPDRS II	UPDRS III	mod. III	PIGD	Extremities	Neck	Hoehn & Yahr	BBS	Golbe	PSP-staging	NNiPPs	FBA	MMSE	PSP-RS	SEADL	MADRS	Verum	Levodopa
PSP1	1	68	174	76	1	5	6	10	14	14	7	0	2	2,5	34	34	2	34	14	29	34	50	13	0	1
PSP3	1	70	172	87	1	24	0	13	19	15	7	2	1	2,5	49	22	2	23	14	29	26	80	10	1	1
PSP4	1	60	176	65	0.5	1	4	7	11	10	14	2	0	3	53	23	2	25	16	29	27	90	15	1	1
PSP5	1	68	168	82	0.5	5					5	/	/	3	33	43	2	/	/	/	43	80		0	1
PSP6	0	70	156	56	0.5	2	2	14	13	26	12	1	2	3	47	24	2	36	13	26	29	70	13	0	1
PSP7	0	60	164	123	1	6	1	8	13	11	6	1	2	2,5	53	17	2	/	16	30	19	90	4	1	1
PSP10	0	66	168	55	1	1	3	10	9	9	5	1	2	2,5	46	18	2	30	13	30	23	90	26	1	0
PSP11	0	68	165	90	0.5	3	2	4		10	5	0	1	2,5	55	17	2	24	11	27				0	1
PSP14	0	65	161	60	0.5	4	1	13	15	13	9	2	2	3	49	26	3	23	15	28	26	80	1	1	0
PSP15	0	65	162	70	0.5	6	2	17	14	14	7	0	1	2,5	54	35	2	26	18	30	31	70	15	1	1
PSP17	1	70	178	74	1	16	3	18		13	15	0	1	2,5	47	35	2	24	15	26				0	0
PSP19	1	69	174	68	1	6	4	21	21	18	10	1	1	2,5	38	31	2	41	9	26	43	60	11	1	0
PSP20	0	73	170	65	0.5	3	5	12		9	6	0	0	2,5	/	22	2	28	7	26				0	1
PSP21	1	70	178	82	1	6	2	12	13	5	3	1	0	2	41	24	2	34	/	28	38	70	5	0	1
PSP22	0	65	168	75	1	2	3	13	17	18	12	1	2	3	49	42	2	22	14	29	38	70	17	0	1
PSP23	0	64	162	68	0.5	22	2	16	13	13	9	0	2	2	42	34	2	26	14	30	39	80	12	0	1
PSP25	1	69	183	102	0.5	9	2	14	13	11	5	0	2	2	48	34	2	25	17	29	30	80	3	0	0

*Whether patients received Rasagiline as participants in the PROSPERA study is marked in the column “Verum” (1 = yes). PSP patients who had Levodopa in their medication regimen are indicated with “1” in the respective column; all IPD patients were given Levodopa as well. In the given collective, there was no significant effect of clinical parameters on performance in vibration effects, nor for Rasagiline within the PSP group*.

Among 20 IPD subjects recruited for a group of postural experiments^1^ [published elsewhere as Ref. (23)], 15 were capable to participate in this study. They were seven females/eight males, 46–73 years of age (median ± SD 70 ± 7.5 with a disease duration of 10 ± 4.5 years), selected from outpatients with known postural instability in the pull test and > = 1 fall/month (reported by patients and family/caretakers where applies). Postural instability and falling remain largely uninfluenced by even otherwise optimized medication [e.g., Ref. (2, 4, 22)], also in a self-induced weight-lifting context [e.g., Ref. (16)]. We aimed at a clinically relevant “normal everyday” state rather than an artificial OFF state (4). Levodopa might even impair certain postural features (16). Therefore, patients were on their regular medication in ON state (recording and evaluation at known best ON after last medication), none had deep brain stimulation. None had agonist-specific side effects. The momentary state of patients’ mobility was assessed just prior to the actual experiment with the Unified Parkinson’s Disease Rating Scale (UPDRS), Hoehn & Yahr stage, and the modified Schwab & England scale for recent capabilities in activities of daily living (SEADL). Additional neuropsychological testing was not performed like in PSP, since it was not in the purview of this study.

From 26 PSP patients in the postural study collective, 17 could perform the weightlifting task (60–73 years old, median ± SD 68 ± 3.6 with a disease duration of 5 ± 6.4 months, nine female, eight male). All but one participated in the PROSPERA study (prematurely ended, randomized double-blinded Rasagiline in PSP, EudraCT 2008-007520-26; no effect on disease progression was shown; Rasagiline or placebo). Clinical testing included (additional to those tested in IPD): PSP Rating Scale (PSPRS), NNiPPS scale (Neuroprotection and Natural History in Parkinson Plus Syndromes), Frontal assessment battery (FAB), Mini-Mental State Examination (MMSE), and Montgomery–Åsberg depression rating scale (MADRS). The in-depth motor and neuropsychiological testing in PSP was part of the PROSPERA evaluation. Most PSP patients received a regular daily dose of Levodopa (typically 100/25 mg three to four times daily), as indicated in Table 1. Medians and SD of clinical scoring where meaningful are given in Table 1.

Control subjects were recruited from among spouses of patients, relatives of the authors, and former university personnel. Among 25 subjects, 16 participated (age 40–73, median 60; 8 females, 8 males). None had history of neurological disorders of any sort or orthopedic disorders requiring surgery or regular medication.

All patients were regularly followed up for 4 years in our outpatient clinic [compare 0–32 months follow-up in Ref. (10)], in which none was re-diagnosed with a different Parkinsonism spectrum disorder. Also none of the control subjects developed any Parkinsonism spectrum disorder.

### Posturography

**Figure 1:**
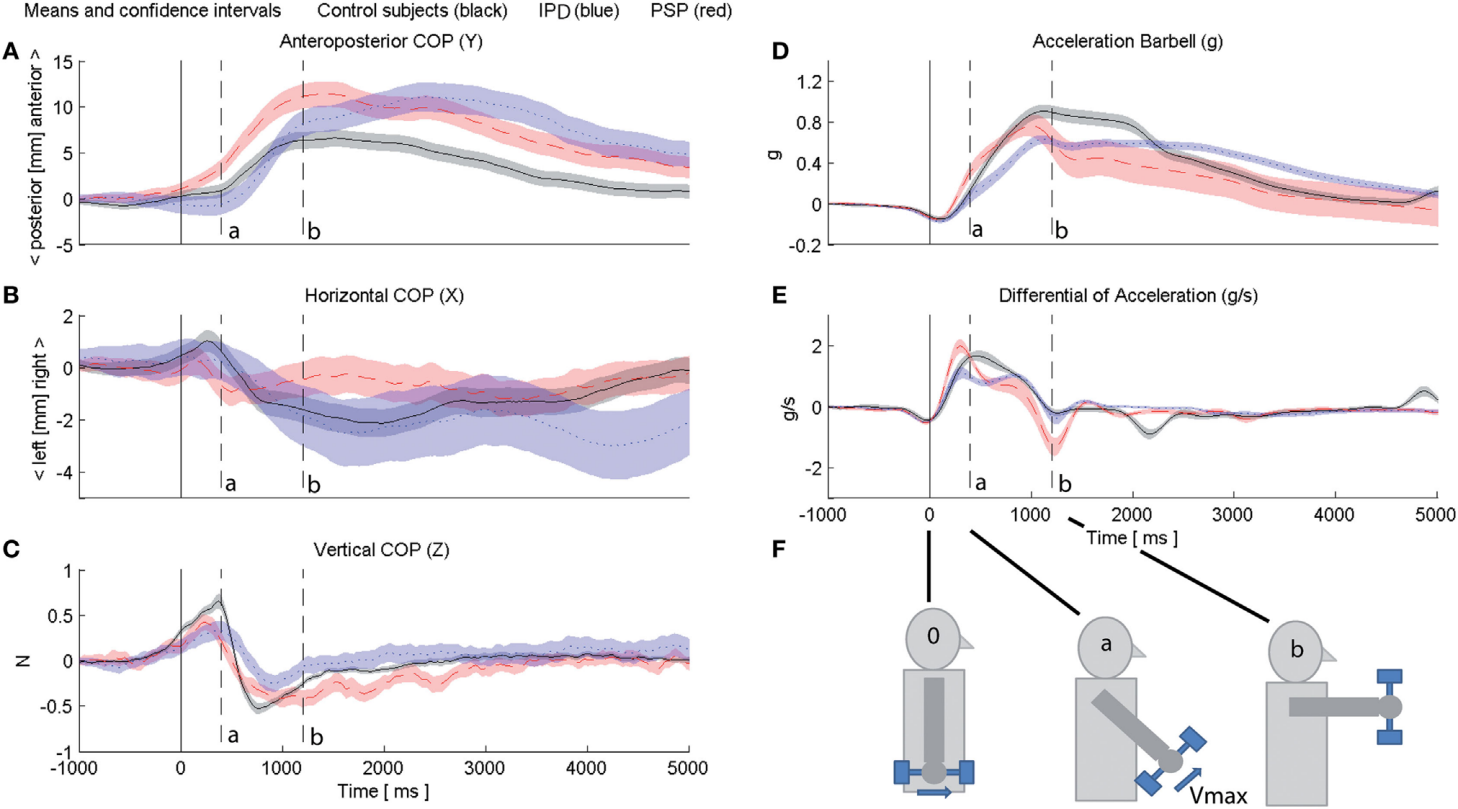
Subjects’ center of foot pressure (COP) depicted for control subjects (black), idiopathic Parkinson’s disease (IPD) (blue), and progressive supranuclear palsy (PSP) (red) with 95% confidence intervals (opaque area of according color) in sagittal **(A)**, lateral **(B)**, and vertical **(C)** directions. Data of all individuals of one respective group are segmented and averaged 1,000 ms prior to the weight lifting onset and 5,000 ms beyond. An accelerometer inside the weight indicated the course of the fast 90° quarter angle motion (position indicated in **(D)** at 0-g hanging down; forward 90° holding applies 1-g gravitational pull); **(E)** depicts weight angular velocity as derivative of **(D)**. Weight lifting onset is at “0.” The peak angular velocity of controls is reached at marker ”a,” the 90° forward position of the weight in controls is reached at marker “b.” Markers “0,” “a” and “b” are depicted continuously throughout **(A–E)**. **(F)** provides a schematic of the barbell motion for visualization.

All subjects stood on an inert recording platform with piezoelectric elements [9281A, Kistler Instrumente AG, Winterthur CH (24)]. The feet were placed together at the heels with the toes spread 30° apart. A PC with Matlab 2007 (The MathWorks Inc., Natick, MA, USA, www.matlab.com) recorded platform signals of anteroposterior (*y*-axis, Figure 1A), lateral (*x*-axis, Figure 1B), and vertical (*z*-axis, Figure 1C) displacement of center of mass (COM) by its surrogate parameter “center of foot pressure (COP)” (24) at 40 Hz together with weight accelerometer data (Figures 1D,E).

### Weight Lifting

Subjects held a barbell with a 3D accelerometer (13) connected to the PC, which also operated the posturography platform. The weight could be adjusted to the highest load subjects could perform the intended task with in one fast motion (14, 18) without exhaustion [all 0.5 or 1 kg, see Table 1; (13, 14, 25, 26)]. All subjects were right-handed subjects and used their dominant right hand.

Trial runs familiarized with the task in five cycles: the weight was held hanging down next to the leg. They lifted it up in a fast quarter circle up to a 90° forward extension, held there for at least 5 s, and finally returned back to the neutral position.

### Barbell Accelerometer

The one-axis accelerometer within the top of the barbell aligned with the plane of the lifting motion equivalent to a position signal (Figure 1D): while hanging down gravitational pull was set at 0 g, in the 90° forward position 1 g was calibrated. From this, the angular rotational velocity was derived (Figure 1E). The onset/initiation of motion (trigger) was defined as the first negative peak of D[Acc] (marker “0” in 1e, propagated to all subplots of Figure 1). Further events were marked “a” (peak angular velocity of control subject grand average at 400 ms) and “b” (1,200 ms, controls reach the final 90° forward position of the barbell) throughout subplots of Figure 1. Figure 1F provides a schematic of the barbell motion for visualization.

### Recording Design

Subjects were given 30 s on the platform to perform at least three weight lifts at their own discretion; they were only given a signal that the platform was recording and when the 30-s time period expired. This procedure was repeated in alternating fashion with eyes open (EO) and eyes closed (EC) 10 times for each condition.

### Data Segmentation, Analysis, and Visualization

Segmentation of trials was performed semi-automatically with Matlab with time range −1,000 ms to + 5,000 ms to marker “0.” Insufficient trials were rejected (such as self-aborted or slow lifts). The trials eligible for further analysis were as follows: IPD median 22 EO (range of accepted trials per subject 5–48)/20 EC (3–47), PSP median 12 EO (8–31)/12 EC (10–32), and controls median 24 EO (3–49)/23 EC (3–47).

Statistics with Microsoft Excel, Matlab, and SPSS 20 used repeated measures ANOVA, Mauchley’s Sphericity/Greenhouse–Geisser correction and Bonferroni *post hoc* for APA (18) analysis in four preset intervals of each 250 ms ranging −1,000 ms to the weight-lifting onset. Statistical significance was set at *p* < 0.05. For CPA analysis, post-trigger, amplitude, and latency of the furthest forward COP displacement for each individual and visual condition were computed. The course of COP displacement was analyzed dynamically in group grand average and its 97.5% confidence intervals (displayed in Figure 1; *p* < 0.05 accordingly).

## Results

For all 4× 250 ms APA intervals before onset, there was no significant difference or interaction between groups and EC/EO conditions [*F*(1,2) = 1.444, *p* > 0.05 for all groups, conditions and latencies]. For CPA grand average, the maximum forward COP displacement in amplitude and latency differed significantly in all groups (*p* < 0.05), irrespective of the EO/EC condition. The IPD peak latency was significantly longer than controls and PSP (*p* = 0.001), which did not differ among each other. Peak amplitudes of PSP and IPD were by far higher than controls (*p* < 0.05; for EO IPD vs. CTR *p* = 0.01, EC *p* = 0.02; for PSP vs. CTR EO/EC *p* = 0.01), but did not differ among each other (*p* > 0.05, but difference in latency as stated above). Within-groups and between-groups where applies, there were no significant effects of the demographic (age, sex) or recorded clinical parameters (scales and scores) for the peak amplitudes or latencies. There was no statistically significant effect of Rasagiline vs. placebo or the presence of Levodopa within the PSP group (all *p* > 0.05).

### Control Subjects

**Figure 2:**
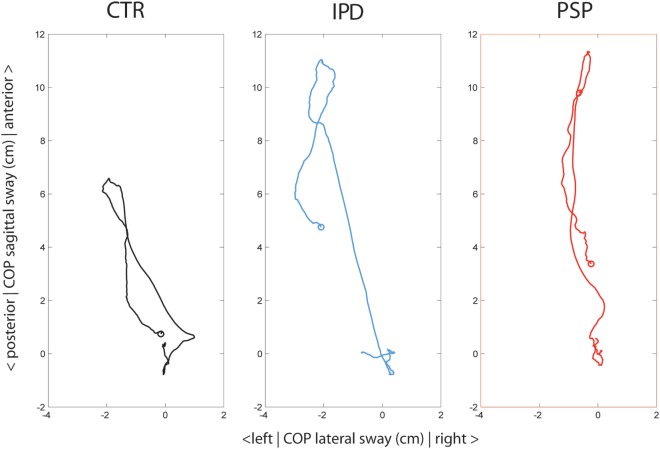
Subjects’ averaged center of foot pressure (COP) depicted from an on-top viewpoint throughout the weight-lifting cycle with the right arm, from −1 s before lift to +5 s after lifting. Groups are color-coded control subjects (CTR, black), idiopathic Parkinson’s disease (IPD) (blue) and progressive supranuclear palsy (PSP) (red). The circle in each graph depicts the final position of COP at + 5 s, at which the weight was still extended 90° forward.

Control COP exhibited an initially slow forward COP displacement during the accelerating phase of the weight lift in the first 400 ms [“0” to “a,” Figure 1; (14)], followed by a faster forward COP during the weight deceleration phase. After the maximum COP forward shift at 1,200 ms with the weight 90° forward (“b”), COP was returned within below 1 cm of its pre-liftoff position during the 5-s weight holding phase (no statistical difference to pre-liftoff within-group *p* > 0.05, Figure 2).

In the lateral direction (Figure 1B), COP shifted ipsilateral right to the weight in the acceleration phase (“0” to “a”), but was compensated rapidly in the contralateral left direction by a fast phase during weight deceleration before reaching its 90° forward arc (“a” to “b,” Figure 1C) and then by a secondary slower body leftward lean once the weight had reached its definite position [“b” up until 3,000 ms, Figures 1B and 2; compare, e.g., Ref. (14)].

### Progressive Supranuclear Palsy

In PSP subjects, peak angular velocity was significantly higher than controls or IPD (*p* < 0.01) and the initial acceleration was significantly faster than controls (*p* < 0.05), indicating a more brisk initial muscle jerk (Figures 1D,E). During the initial displacement, PSP started to shift their COP further forward (*p* < 0.05). At arrival of the weight at 90° forward (marker “b”), PSP used significantly more active braking in order to stop the weight at its intended 90° forward position (Figures 1D,E). Throughout the following holding period, PSP maintained their COP further forward compared with controls and even at 5,000 ms after the weight lift, their COP did not reach within 2 cm of the initial neutral position.

In the lateral direction (Figures 1B and 2), PSP lacked the compensatory leftward body lean contralateral to the extended weight, especially 1,000–3,000 ms (to controls *p* < 0.01, to IPD *p* < 0.05). Their forward excursion was a pure anterior COP shift. During the return of COP, PSP displayed high-frequency oscillations of the body in both lateral (Figures 1B and 2) and vertical planes (Figure 1C), which were not observed in controls or IPD. In the 5-s holding period, COP returned within 4 cm of original position.

### Idiopathic Parkinson’s Disease

Idiopathic Parkinson’s disease was the only group to shift COP backwards at the onset of weight shift, previously described as overshooting “APA” or early CPA (14, 20). The grand average peak excursion of this motion did not reach significant difference controls at its peak latency (*p* = 0.06, Figure 1A). IPD kept pushing their COP forward beyond reaching the weight 90° forward position (“b”). This slow exaggerated forward push was significantly later than PSP by around 1.4 s, but not significantly different in amplitude. After the 3-s mark IPD COP displayed a slow-frequency tumble in both anteroposterior and lateral directions toward neutral position, without reaching within 4 cm of COP baseline in the 5-s holding period.

## Discussion

Progressive supranuclear palsy and IPD displayed postural distinct abnormalities during rapid lifting of a light object, a frequent task in daily life, e.g., placing a jar on a shelf (13). Healthy subjects compensated the asymmetric unilateral weight shift already during peak object velocity (14, 17) and their COP excursion was fully recompensated.

A considerable preemptive backward lean of COP requires 5–10% of body weight in this task (27). We could demonstrate how even smaller weights could unmask disease-specific deficits in IPD (14, 20) and in PSP with COP posturography, which were not known to date. Only a small late APA component became apparent in IPD at liftoff. Due to its purview on small self-triggered weight displacement this study cannot draw conclusions about APA differences in PSP vs. IPD.

The visual conditions EO/EC effects did not reach significant levels, very likely due to the deeply ingrained and overlearned characteristic of this daily task, the small weight and the postural challenge being so low that it did not push even PSP near their limits of stability [(22) and references therein]. That difference would amplify under greater and particularly multimodal sensory challenge (24), which emphasizes the functional relevance of the postural pathology observed here occurring particularly within limits of stability (22).

### Idiopathic Parkinson’s Disease

Idiopathic Parkinson’s disease presented pathology expected from advanced-stage subjects with slow angular velocity, due to predominant extremity rigor. IPD COP displaced backwards as a late motion-onset APA, disproportionately overscaled to the small weight (14, 20), yet also functionally insufficient: IPD COP overshot controls at 90° extension (“b,” Figure 1A) and continued to a delayed COP forward lean around 3 s after liftoff. In conjunction with stooped posture, this corresponds to the frequently reported delayed falling pathology in IPD during a lifting task. In this study we observed this effect occurring already with very light weights, which have also been described to result in falls infrequently and which is not described in the literature as a particular deficit. Lateral oscillations in IPD with slow wavelengths around 3 s (0.3 Hz), and large amplitude were pronounced in the return of COP from peak displacement, which may be explained by a combination of postural body harmonics influenced by rigidity plus oscillations in the central neural network (22).

### Progressive Supranuclear Palsy

Progressive supranuclear palsy presented higher acceleration and deceleration (Figures 1D,E) than controls, which indicates deficits in force scaling, effects similar to those found in young children (28). This has not been observed in PSP before and whether this only applies to a postural context with self-triggered motion, or whether it is a general feature of the disease, should be further investigated. Oscillations have not been seen as a particular PSP feature among the same identical patients during passive surface displacement (see text footnote 1) or passive neck vibration (23).

The rapid forward overshoot of COP may be due to either (a) insufficient APA/CPA for the rapid weight shift (14, 20), and/or (b) a compensatory central rescaling of stability boundaries forward protecting against backward falls, which would restrict backward motion even when warranted as part of purposeful APA/CPA. The forward displacement was more strictly aligned sagittally than in the other groups, possibly as a sign of the proprietary PSP axial rigidity (6). Despite similar displacement amplitudes as IPD, PSP patients do not report problems in this context, possibly due to the short and rapidly reversed excursion. A similar fast-forward overshooting and slow return was found in young children <6 years (29), where (similar to excessive force scaling) certain mechanisms have not yet matured; in PSP these effects might be equivalent to disease-specific system devolution. Leaky CPA compensation to slow frequencies and backward direction in comparison to the possibly intact fast CPA as suggested above may be a disease-specific feature, warranting further investigation.

Beyond these similarities, the COP returning motion exhibited high-frequency oscillations in the lateral and axial planes at wavelengths around 0.75 s (1.3 Hz, compare 0.3-Hz oscillations in IPD). In an active tilting platform experiment up to 2 Hz (see text footnote 1), PSP were characterized by frequency-dependent excessive gain responses to passive tilting especially for the upper body segment, but particularly no central system oscillations were reported. Also in the lateral plane, these appear to be specific to the self-induced motion paradigm.

The description of these particular deficits suggests that they may be trained and hopefully compensated or at least slowed in progression by physiotherapeutic means. Practically, ambulatory PSP and late-stage IPD fallers should consider exercises with small weights to adapt and compensate overshooting and wrong directionality in a relatively safe task, since it could be performed by even severely affected PSP patients in this study. These routines can be included easily in daily training. Challenges can be added by increasing weights or withdrawing sensory qualities. The task is naturally overlearned with apparent practical everyday use. Its training may also facilitate compensatory strategies in other postural contexts. Studies should evaluate whether there is a benefit of training these tasks with posturographic analysis and with clinical rating scales over several months to detect possible improvements or extension of ambulatory capabilities.

## Conclusion

Self-triggered lifting of small weights is pathologically impaired in PSP and IPD. PSP shows exaggerated and oscillatory body motion, similar to naturally evolving behavior in young children but instead possibly due to pathological postural system devolution. IPD present a slow two-phased slow forward overshooting of the body, which may contribute to falling. The two different pathological postural responses are suggested to be targeted with physiotherapy training of the respective deficits.

## Ethics Statement

This study was carried out in accordance with the recommendations of decision 142/04 of the LMU ethics committee with written informed consent from all subjects. All subjects gave written informed consent in accordance with the Declaration of Helsinki. The protocol was approved by said committee.

## Author Contributions

SK and KB composed the manuscript. SK, LD, KM, AP, and AS performed the experiments. SK, AP, and SL performed clinical assessment. SK and AS performed the statistics. SK and AA performed data analysis and visualization.

## Conflict of Interest Statement

The authors declare that the research was conducted in the absence of any commercial or financial relationships that could be construed as a potential conflict of interest.

## Footnotes

^1^Kammermeier S, Maierbeck K, Dietrich L, Plate A, Lorenzl S, Singh A, et al. Qualitative postural control differences in idiopathic Parkinson’s disease vs. progressive supranuclear palsy with dynamic-on-static platform tilt. *Clin Neurophysiol* (Under review).
